# Partial Exsection of Both Upper Jaws for Enchondroma

**Published:** 1875-07

**Authors:** 


### ARTICLE IV.

*Partial Exsection of both Upper Jaws for Enchondroma.*

Dr. Porter reported the case and showed the specimen at a meeting of the Boston Society for Medical Improvement.

The patient, a gentleman aged fifty-one, of excellent health, first noticed nine years ago a congested condition around the two central incisors of the upper jaw. These were "pivot" teeth, inserted twenty years before, and as they often became loose, he had been in the habit of replacing the pivots by the ends of matches. There finally resulted a necrosis of the alveolar process around these teeth, which gradually exfoliated. Soon a defined tumor commenced in the above locality, which has been growing for three years. It has been for the most part painless, but has doubled in size within six months. The patient's family history shows no record of tumor.

The tumor now involves the upper alveolar arch nearly symmetrically on both sides, from the first molar tooth on one side, to the corresponding tooth on the opposite side, extending nearly up to the infra-orbital foramina, pushing upwards and forwards the nose and upper lip, producing a very considerable deformity.

The operation for the removal of the growth was performed with ether. A Y-shaped incision was first made, the long arm of the Y cutting through the median line of the lip and the short arms extending, one into each nostril. The coronary artery was controlled by a deep temporary suture in each flap. The soft parts on both sides were then dissected up as high as the level of the infra-orbital foramen, and backwards to the tuberosity of the superior maxilla. The hæmorrhage was readily controlled by sponges stuffed into the wound. The second molar tooth on each side was then drawn, the mucous membrane covering the hard palate cut transversely, and the alveolar process and hard palate sawed through from side to side, and upwards quite into the antra. The anterior wall of the antrum and the nasal process on each side were cut through by a few quick strokes with mallet and chisel, the septum nasi severed at the union with the palate process, and the tumor easily depressed and removed entire. A number of large vessels required ligature, though much less blood was lost than would be supposed from the extensive laceration of the soft parts. The wound was brought together by sutures. The pulse remained good throughout.

The tumor, examined, by Dr. R. H. Fitz, proved to be enchondroma. The patient made a rapid convalescence, and returned home on the eighth day after the operation.

In four months from the first operation, he noticed a return of the growth on each side of the nose, and when seen two months later, the tumor of the right side was about the size of an English walnut, involving the nasal process of the superior maxilla; that of the left side was similarly situated but less in extent and size, being as large as a chestnut.

Before the operation for the removal of the tumor was commenced, the patient was etherized and tracheotomy was done. Sponges were then introduced into the pharynx to prevent bleeding into the throat. As in the first operation, a Y-shaped incision was made through the middle of the lip upwards into each nostril. The cheeks were freely dissected up, on the right side as high as the inner commissure of the eyelids, and on the left to a level with the floor of the orbit. The bony attachments were then cut with chisel and mallet, the whole nasal process being removed on the right side and nearly the whole on the left. The wound was closed as before. The tracheotomy-tube was left in for a few hours, until all bleeding had ceased, when it was removed and the wound was closed. The patient rallied well from the operation and made a more rapid recovery than on the first occasion, returning home on the seventh day. The tumors, examined by Dr. Fitz, were like the first, but richer in cells.

Dr. Porter said he wished to call attention to but one practical point in the first operation, and that was the small extent of the incision. The Y-shape of the cut through the median line of the lip, into each nostril, made the circumference of the nostril available and gave an abundance of room for the operation; the resulting cicatrix was very small and entirely covered by the mustache. This method of incision, moreover, results in no paralysis of any of the facial muscles. In the second operation, tracheotomy before hand did away with the constant sponging of the throat consequent upon the character of the operation, and also with the danger from blood in the trachea.

Enchondroma of the jaws is a rare disease, and particularly so that of the upper jaw. Mr. Paget says of enchondroma, "On the jaws these tumors are, I believe, very rare. I know but one specimen on the upper jaw alone, reported in "Guy's Hospital Reports." In the *Dublin Quarterly Journal of Medical Sciences* for November, 1857, Dr. Oscar Heyfelder, of Munich, reports only eight cases of enchondroma out of four hundred and fifty cases of diseases of the superior maxilla, and this number includes the one mentioned by Mr. Paget. In the Records of the Massachusetts General Hospital is a case of enchondroma of the lower jaw removed by Dr. H. J. Bigelow. Heath (Diseases of the Jaws) says, "Cartilaginous tumors of the upper jaw are of uncommon occurrence," and mentions two cases not referred to above. The size of the tumors varied from that of a hen's egg to that of a man's head. In two cases, the nasal process only was removed; in another the nasal process and anterior wall of antrum; one partial resection (not defined,) and one total removal of superior maxilla; in three cases no operation on account of extent of disease. I can find but few reported cases of partial or total exsection of both upper jaws. Partial resection of both bones was done in 1824 by Roger, afterwards by Liston and Dupuytren; but the first total removal of both upper jaws was done by Dr. J. F. Heyfelder, of Munich, who did it three times in 1845, and afterwards in 1850 and 1852.—*Boston Med. and Surg. Journal.*

---

